# Geriatric assessment for oncologists

**DOI:** 10.7497/j.issn.2095-3941.2015.0082

**Published:** 2015-12

**Authors:** Beatriz Korc-Grodzicki, Holly M. Holmes, Armin Shahrokni

**Affiliations:** ^1^Memorial Sloan Kettering Cancer Center, Weill Cornell Medical College, New York, NY 10065, USA; ^2^Division of Geriatric and Palliative Medicine, The University of Texas Health Science Center at Houston, Houston, TX 77030, USA

**Keywords:** Geriatric oncology, geriatric assessment (GA), frailty

## Abstract

The world is experiencing aging of its population. Age-specific incidence rates of cancer are higher and cancer is now recognized as a part of aging. Treating older patients can be challenging. The clinical behavior of some tumors changes with age and the aging process itself brings physiological changes leading to decline in the function of organs. It is essential to identify those patients with longer life expectancy, potentially more likely to benefit from aggressive treatment *vs.* those that are more vulnerable to adverse outcomes. A primary determination when considering therapy for an older cancer patient is a patient’s physiologic, rather than chronologic age. In order to differentiate amongst patients of the same age, it is useful to determine if a patient is fit or frail. Frail older adults have multiple chronic conditions and difficulties maintaining independence. They may be more vulnerable to therapy toxicities, and may not have substantial lasting benefits from therapy. Geriatric assessment (GA) may be used as a tool to determine reversible deficits and devise treatment strategies to mitigate such deficits. GA is also used in treatment decision making by clinicians, helping to risk stratify patients prior to potentially high-risk therapy. An important practical aspect of GA is the feasibility of incorporating it into a busy oncology practice. Key considerations in performing the GA include: available resources, patient population, GA tools to use, and who will be responsible for using the GA results and develop care plans. Challenges in implementing GA in clinical practice will be discussed.

## Introduction

With the increase in the aging population and in life expectancy oncologists are aggressively treating patients into their 80s and even 90s. Patients who previously would have received only symptomatic treatment are now being treated with curative intent without hesitation. The world is experiencing an aging of its population. Age-specific incidence rates of cancer are higher^1^, and cancer is now recognized as a part of aging. Cancer is diagnosed at a higher rate (53%), accounts for a higher percentage of survivors (59%) and results in more deaths among individuals aged 65 years and older (68%) compared with younger adults^2^. This trend implies that the burden of cancer in our population is expected to rise along with the need for specialized services and programs to address those needs.

With a paucity of data to make evidence-based decisions in this population, clinicians need to extrapolate from studies done with a much younger cohort. However, treating patients in their 80s is not the same as treating patients in their 50s or 60s. The clinical behavior of some tumors changes with age. Some become more aggressive due to a high prevalence of unfavorable genomic changes or resistance to chemotherapy. For example, when compared to middle-age individuals treated for acute myelogenous leukemia (AML), older adults experience shorter survival. Age-related differences in tumor biology and resistance to therapy are a major factor influencing treatment outcomes of AML in older adults^3^^,^^4^. Other cancers become more indolent, like breast cancer, due to an increased prevalence of hormone-receptor rich tumors and endocrine senescence^5^.

The aging process itself brings physiological changes leading to a decline in organ function. For example, kidney function decreases with age, pulmonary compliance declines, as does bone marrow cellularity and reserve. The remodeling of physiological reserve or “homeostenosis” is influenced not only by genetic factors but also by environmental factors, dietary habits, and the interaction of comorbidities as well as social conditions. Chronological age differs from functional age, and this difference due to the uniqueness of each patient needs to be captured and integrated in the decision-making process of cancer treatment. It is essential to identify those patients who are fitter and potentially more resilient, because they are more likely to benefit from aggressive treatment, as opposed to patients that are more frail and vulnerable to adverse outcomes. This review will describe the clinical characteristics of frailty in the older adult, how to assess geriatric syndromes through a comprehensive geriatric assessment (GA), the association of GA results with cancer-related outcomes and how to integrate GA into the daily oncology practice.

## The fit *vs.* frail older adult

### The frailty syndrome

A primary determination when considering appropriate therapy for an older patient with cancer is a patient’s physiologic age, rather than chronologic age. In order to differentiate amongst patients of the same age, it can be useful to determine if a patient is fit or frail. Fit older adults have few comorbidities, no functional deficits, few (if any) geriatric syndromes, and generally are considered appropriate for the same therapies used in younger adults^6^. Frail older adults, in contrast, have multiple chronic conditions, difficulties maintaining independence, and geriatric syndromes. They may be more vulnerable to toxicities from therapy, and may not have substantial lasting benefits from therapy, due to multiple factors that pose competing risks for morbidity and mortality^7^^,^^8^.

Frailty is a syndrome of advancing age characterized by immune dysregulation, chronic inflammation, sarcopenia, increased cellular senescence, and a loss of resilience^9^. This leads to a substantial loss of reserve that may become apparent in response to physiologic or psychologic stressors^10^. Patients with the clinical picture of frailty have surrogate biomarkers including elevated cytokines and chemokines, such as IL-6. Frail individuals have also been found to have reduced levels of insulin-like growth factor 1, low levels of dehydroepiandrosterone sulfate and leptin, as well as abnormal white blood cell distributions. Thus, multiple biologic abnormalities are implicated in the development of frailty^9^. In addition, genetic variations in nuclear and mitochondrial DNA have been associated with frailty. Frailty has not been associated with changes in telomere length, perhaps because frailty is largely related to changes in skeletal muscle, in which there is little change in telomere length over time^11^. Epigenetic variations have also been investigated, with frail individuals having higher global levels of DNA methylation^12^.

Clinically, frailty is characterized by a loss of organ function, vulnerability to stressors, and poor physical function^13^. One hypothesis is that aging itself can be a trigger for the frailty syndrome; other changes such as malnutrition, cognitive impairment, social isolation, and immobility can potentially trigger frailty, ultimately resulting in a syndrome manifested by decreased physical function^14^.

Main clinical features of frailty:

- Decreased functional reserve.
- Impairment or dysregulation in multiple physiological systems.
- Reduced ability to regain physiological homeostasis after a stressful and destabilizing event.

### Diagnosis of frailty

Several different tools have been developed to identify frail adults. The most commonly used are the frailty phenotype (FP) and the frailty index (FI). The FP was developed using population-based data from the Cardiovascular Health Study^15^^,^^16^. The components include five criteria: weight loss, low physical activity, weak grip strength, slow gait speed, and exhaustion. **Table 1** shows the definitions for each of these measures.

**Table 1:** Frailty phenotype (FP)^15^^,^^16^

Characteristics of frailty	Cardiovascular health study measure
Unintentional weight loss	At the initial visit: lost >4.5 kg in the prior year; at follow-up visit: loss of 5% body weight from previous year to current weight
Grip strength	Grip strength in the lowest 20% stratified by gender and body mass index (BMI): women, (I) ≤17 kg for BMI ≤23; (II) ≤17.3 kg for BMI 23.1-26; (III) ≤18 kg for BMI 26.1-29; (IV) ≤21 kg for BMI >29; men, (I) ≤29 kg for BMI ≤24; (II) ≤30 kg for BMI 24.1-26; (III) ≤30 kg for BMI 26.1-28; (IV) ≤32 kg for BMI >28
Exhaustion	Exhaustion by self-report, based on how the patient felt the previous week: either feeling that everything the person did was an effort or feeling unable to get going
Slow gait speed	Based on measured time to walk a distance of 15 ft
Low physical activity	Lowest 20% of Kcals/week: men <383 Kcals/week; women <270 Kcals/week

Frailty is present, if ≥3 criteria are present; intermediate or pre-frail, 1 or 2 criteria present; older individuals with none of the above five criteria are classified as non-frail or fit.

The FI is a 70-item tool based on the concept of accumulation of deficits being a measure of frailty^17^. The FI was developed from the Canadian Study of Health and Aging, a 5-year prospective cohort study. It is a count of deficits that includes presence of comorbidities as well as severity of conditions, functional deficits on activities of daily living, and findings from clinical and neurologic examinations. Severity is indicated for applicable conditions by using 3 or 4 values between 0 and 1, rather than a simple dichotomized presence/absence. The deficits are added and divided by the total (70 items) to get the FI score. Relative fitness or frailty is conceptualized as the difference between an individual’s FI score compared to the average score for people at that age^18^. The FI was considered cumbersome to use in clinical practice. As a result, the Clinical Frailty Scale was developed and validated by comparing responses to the values in the FI. Clinicians rate a patient’s fitness or frailty on a scale of 1 to 7, based on the accumulation of deficits and functional impairments (**Table 2**).

**Table 2:** Clinical frailty scale^18^

Scale	Fitness of frailty
Category 1	Very fit: robust, active, energetic, motivated; the fittest group for their age
Category 2	Well: no active disease symptoms, but less fit than people in category 1
Category 3	Managing well: disease symptoms are well controlled compared with those in category 4
Category 4	Vulnerable: not dependent on others but symptoms limit activities, they are “slowed up”
Category 5	Mildly frail: limited dependence on others for some instrumental activities of daily living
Category 6	Moderately frail: help is needed with both instrumental and basic activities of daily living
Category 7	Severely frail: completely dependent on others for self-care or terminally ill

### Prognostic significance of frailty

Based on a systematic review, the prevalence of frailty according to FP in the general population is 14%, and according to FI is 24%^19^. Frailty was more prevalent in women and in minorities. While frailty may vary substantially based on the tool used to diagnose the syndrome, regardless of the diagnostic method used, the frailty syndrome is associated with substantial morbidity and mortality^20^. Frail individuals have a 15% (based on FI) to 50% (based on FP) increase in mortality risk^19^. In a secondary analysis of the Survey of Health, Ageing and Retirement in Europe, 8 different frailty indices were operationalized and, depending on the measure used, the prevalence of frailty ranged from 6.1% to 43.9%. The scales differed with respect to their discriminatory effect for 2-year mortality, with an area under the curve (AUC) ranging from 0.70 to 0.77^21^.

### Frailty and cancer

Frailty is increasingly recognized to be of importance in cancer. There remains a question whether cancer itself contributes to the development or acceleration of the frailty syndrome, given the commonalities between the two disease states. Both are characterized by immunologic dysregulation and dysfunction, sarcopenia, and cachexia. In addition, frailty is a potentially important outcome in cancer survivors, as a consequence of cancer treatment^10^. Older cancer survivors have an odds of developing frailty of 1.46 (95% CI, 1.29-1.65) compared to older persons without a history of cancer^22^.

Frailty has been associated with increased mortality in older patients with cancer. However, the definition of frailty in such studies has largely been based on the results of GA. Very few studies have used the FP or FI for the evaluation of outcomes in patients with cancer. Frailty according to the FP was associated with an increased postoperative complications and postoperative mortality in gynecologic oncology patients and in patients undergoing colorectal surgery^23^^,^^24^.

Evaluating frailty in older patients with cancer has several potential goals. Understanding the biology of aging and frailty could contribute to an understanding of the biology of cancer and aging. Detecting frailty may lead to the identification of patients at greater risk of adverse outcomes and may provide information for diagnostic and treatment planning. However, frailty may not be an adequate construct; a comprehensive evaluation that results in actionable information may necessitate a more complete GA^25^.

## Geriatric assessment (GA)

GA is a multi-dimensional, interdisciplinary evaluation used primarily by geriatricians for several potential purposes. GA is used to determine physiologic as opposed to chronologic age. The evaluation is also used to determine whether a patient is fit, vulnerable, or frail. GA is used as a tool to guide future diagnostic and therapeutic interventions, i.e., to determine any reversible deficits in older persons and devise treatment strategies to eliminate or mitigate such deficits. GA is also used to assist in treatment decision making by clinicians by helping to risk stratify patients prior to potentially high-risk therapy^26^.

Ultimately, there is no standard definition of a GA. However, a position paper by the International Society of Geriatric Oncology (SIOG) has helped to clarify necessary elements of GA and provide potential guidance as to the tools that could be used for each element^26^. A GA generally includes validated tools that assess several domains important in determining physiologic age: comorbidity, functional status, physical performance, nutritional status, polypharmacy, social support, cognition, and psychological status (depression and anxiety). These domains are shown in **Table 3** along with examples of validated tools to measure those domains.

**Table 3:** Domains in geriatric assessment and examples of tools used for each domain

Domain	Tool
Social status and quality of life	Medical outcomes survey^27^
Comorbidity	Charlson Comorbidity Index^28^; Cumulative Illness Rating Scale-Geriatrics^29^
Functional status	Activities of daily living^30^;Instrumental activities of daily living^31^
Physical function	Timed up and go^32^; short physical performance battery^33^; grip strength; falls and fall risk
Cognition	Mini-Mental State Examination^34^; Montreal cognitive assessment^35^; Blessed Orientation-Memory-Concentration (BOMC) test^36^; Mini-Cog^37^
Nutrition	Body mass index; unintentional weight loss; Mini Nutritional Assessment^38^
Medication management & polypharmacy	Use of inappropriate medications (such as the beers list or screening tool for older persons’ prescriptions)^39^; number of medications
Psychological status	Geriatric depression scale^40^; hospitalized anxiety and depression scale^41^; patient health questionnaire-9^42^

### Assessment of comorbidities

The incidence of pathology increases as people age. The presence of multiple chronic diseases or comorbidities represents a major difference between younger and older cancer patients. Frequent comorbidities in the elderly such as cardiovascular disease, hypertension, diabetes or dementia influence the management of cancer. Comorbidities may increase the risk of complications, modify cancer behavior, or mask symptoms with subsequent delays in cancer diagnosis. On the other hand, cancer treatment may worsen comorbidities or increase the frequency of drug interactions.

Comorbidity burden is often measured using standardized indices. Commonly used indices are the Charlson Comorbidity Index (CCI)^28^ and the Cumulative Illness Rating Scale-Geriatrics (CIRS-G)^29^. The CCI is based on the 1-year mortality of patients admitted to a medical hospital service. It is a simple instrument, with rating criteria well defined; it was adjusted for age and can be used for large cohort studies. However, it may under-detect non-lethal endpoints. The CCI has been validated in older cancer patients^43^. The CIRS-G is more comprehensive but may over-detect minor problems and it is quite complicated to rate. The geriatric version of the CIRS was designed for the elderly population and details several geriatric problems in the list.

Available evidence and clinical experience would support evaluating major comorbidities as a method for identifying frail older adults during a pre-treatment assessment. In a US population of older breast cancer patients, 13 individual comorbid conditions were associated with decreased overall survival and increased mortality^44^. A recent review of the impact of comorbidity on cancer survival showed that both treatment effectiveness and compliance appear compromised among cancer patients with comorbidity^45^. Comorbidities influence the patient’s life expectancy independently of the cancer^46^. In the NCCN Clinical Practice Guidelines in Oncology (NCCN Guidelines^®^ Senior Adult Oncology 2014, the approach to decision making in the older adult starts with the question: “*Does this patient have a life expectancy that puts him/her at moderate/high risk of dying or suffering from this cancer during his/her life expectancy?*” If the answer is “no”, symptom management and supportive care are recommended.

### Cognitive assessment

Cancer patients with cognitive dysfunction represent a new challenge for oncologists. After age 65 the risk of developing Alzheimer’s disease doubles about every 5 years. By age 85, 37% of all people will have some signs of the disease^47^. The increased rate of dementia in the elderly converges with the higher likelihood of developing cancer. Patients with cancer/dementia overlap are often diagnosed later in the disease process, screening is less standardized and adherence with treatment is often difficult. Many patients with mild dementia do not appear to be impaired. But, impaired cognition can result in significant difficulties in understanding and remembering treatment instructions, delayed diagnosis of complications and less compliance with oral therapies and supportive treatments. Therefore, initial cognitive status could influence the choice of treatment and the modality of administration.

There are several instruments validated for cognitive screening (**Table 3**). The Mini-Mental State Examination (MMSE)^34^ is one of the most widely used screening tools covering multiple domains such as: orientation, memory, attention, calculation, language, and constructional ability. The Montreal Cognitive Assessment (MoCA) is a more sensitive test as it was designed as a rapid screening instrument for mild cognitive dysfunction^35^. It was found to provide additional information over the MMSE in brain tumor patients^48^. The Blessed Orientation-Memory-Concentration Test^36^ is a brief, 6-item scale frequently used in the geriatric oncology literature as a component of the Cancer and Aging Research Group toxicity tool^49^. The Mini-Cog assessment instrument^50^ is a brief test for discriminating demented from non-demented persons in a community sample of culturally, linguistically and educationally heterogeneous older adults. It requires minimal training to administer so it can be readily incorporated into general practice.

The ability of patients to decide on a course of therapy in concert with the oncologist is critically important. Many oncologists are conflicted as to whether true informed consent for treatment can be obtained from older cancer patients when their cognitive abilities are impaired or unclear. It is imperative that health care providers that care for older cancer patients be able to assess cognitive function and understand the implications of cognitive impairment on decision-making. Furthermore, they should address the potential for treatment-related cognitive decline and facilitate patient-centered, shared decisions.

### Medication management and polypharmacy

Pharmacotherapy of the elderly is very complex due to age-related physiologic changes, multiple comorbidities and multiple medications. In addition, cognitive impairment, functional difficulties, as well as caregiver issues play a large role in errors and compliance. Age-related physiologic changes and disease-related changes in organ function affect drug handling (pharmacokinetics) and response (pharmacodynamics) (**Table 4**) with a significant impact on prescribing. As people age, they accumulate chronic conditions, and standard medical care of these conditions involves multiple drugs. In addition, cancer patients usually take multiple medications, not only for the treatment of the cancer, but also for supportive care and the management of symptoms related to therapy-induced toxicity^52^.

**Table 4:** Age-related changes in pharmacokinetics and pharmacodynamics^51^

Pharmacokinetics	Age-related changes	Clinical consequences
Absorption	Changes in gastric motility and bowel transit time; changes in blood flow to the gut	None described
Distribution	Decrease in lean body mass; decrease in total body water; increase in body fat; decrease in serum binding proteins: albumin decreases	Decrease volume of distribution of water soluble drugs with higher blood levels; increase volume of distribution of fat soluble drugs with increased half-life; decrease binding of acidic drugs to albumin with elevation of free-drug level even if the total concentration of the drug is decreased
Metabolism	Reduced liver mass and reduced hepatic blood flow; reduced enzyme activity of the cytochrome p450 system	Reduce rate of drug metabolism; increase variability in drug bioavailability
Elimination	Reduced renal blood flow and renal mass; sclerotic changes of the glomeruli; infiltration of chronic inflammatory cells and fibrosis in the stroma	Loss of glomerular filtration capacity; decrease in concentrating and diluting ability; decrease elimination; increase half-life
	Reduced sensitivity of arterial pressure receptors with decreased baroreceptor reflex response	Postural hypotension; post-prandial hypotension
	Decreased responsiveness of B-adrenergic receptors	Limits heart rate and contractile response to stress
	Decreased sensitivity of respiratory centers to hypoxia and hypercapnia	Delayed and/or diminished ventilatory response
	Loss of neuronal substance, decreased synaptic activity, impaired glucose metabolism in the brain and more readily penetration of drugs in the central nervous system (CNS)	Higher susceptibility and exaggerated response to drugs that interact with the peripheral and central nervous system

Potentially inappropriate medications (PIM) are medications that pose more risk than benefit to the patient either because they are ineffective, they pose unnecessary risks, or there are safer alternatives available. A consensus guideline known as the Beers criteria, first published in 1991 and last updated in 2015 provides a list of drugs that a panel of experts thought to be particularly problematic for older patients^39^. The prevalence of polypharmacy and PIM in older adults with newly diagnosed cancer was 80% and 41%, respectively, which, in turn, led to adverse drug events and increased morbidity^53^. Polypharmacy and non-adherence are well documented problems among elderly patients^54^. With the development of oral anticancer drugs, adherence has become an important factor in the success or failure of treatment.

### Social issues and quality of life

Social support has a substantial impact on cancer. Evidence in breast cancer patients suggests that low social support is associated with development and progression of cancer^55^. Once diagnosed, cancer has a substantial impact on quality of life and on social function at any age. Older patients with cancer may have additional challenges in the need for caregivers, transportation, and home care to be able to safely undergo cancer therapy. Social isolation and low levels of social support have been associated with an increased incidence of cancer as well as higher mortality risk in patients with cancer^56^^,^^57^. Increased social isolation is also a risk factor for poor tolerance of adverse effects of cancer treatment^58^.

### Assessment of physical function

Oncologists usually measure physical function using subjective scales such as the Eastern Cooperative Oncology Group (ECOG) or Karnofsky performance status scales. Physical function can also be assessed by objective measures of performance, including gait speed, grip strength, balance, and lower extremity strength, which are more sensitive and shown to be associated with worse clinical outcomes^59^. A commonly used test for gait speed is the timed up and go, which is brief and easy to implement in clinical settings^32^. The Short Physical Performance Battery is another tool that assesses gait speed, in addition to lower extremity strength and balance^33^. Gait speed is an important indicator in older persons, as it has been shown to be an independent predictor of mortality across numerous population-based studies^60^. Grip strength is also important to assess in cancer patients and is relatively quick and easy to do; however, the availability of a hand-held dynamometer may be a barrier. Grip strength is a measure that correlates with sarcopenia, and has been shown to be associated with adverse outcomes in patients with cancer^61^^,^^62^, and associated with mortality in general populations^63^^,^^64^.

Falls are major events and major health concerns in the older population since they are related with the person’s ability to live independently. More than one third of persons aged 65 years or older fall each year, and in half such cases the falls are recurrent^65^. They are typically multifactorial and due to intrinsic factors (e.g., visual impairment, muscle weakness, poor balance, orthostasis), extrinsic factors (e.g., polypharmacy, medication side effects) or environmental factors (e.g., loose carpets, poor lighting, etc.). Falls need to be thoroughly evaluated using a multidisciplinary approach (physical therapy, occupational therapy, home safety, medication evaluation, evaluation for cataracts, etc.) with the goal to minimize the risks without compromising functional independence. The Tinetti Gait and Balance Scale is a rapid, reproducible assessment tool for the evaluation of fall risks, gait and balance^66^. The test is scored on the patient’s ability to perform specific tasks. Time to complete is 10-15 min and inter-rater reliability was found to be over 85%.

There is a particular need for falls screening among older patients with cancer^67^. This group of patients has additional risk factors for falls such as toxicity from cancer treatments or brain metastatic disease^68^^,^^69^. The presence of falls was shown to be associated with an increased risk of serious chemotherapy toxicity in older cancer patients^49^. Chemotherapy-induced peripheral neuropathy was shown to be associated with falls (11.9%) and functional impairment (26.6%) in a cohort of  421 patients^70^. In one study, only 10% of older cancer patients with cancer who self-reported a recent fall had appropriate medical record documentation showing the need to increase oncologists’ awareness of falls prevalence and consequences in order to provide timely interventions such as referral to physical therapy for rehabilitation or exercise programs^71^.

### Functional status

An assessment of functional status includes daily living dependence scales and determining whether a patient needs any assistance on instrumental activities of daily living (IADLs) or activities of daily living (ADLs). IADLs generally refer to tasks that are needed to live independently in the community and include shopping, transportation, using the telephone, managing finances, medication management, cooking, cleaning, and laundry^31^. ADLs are basic self-care skills needed in order to live independently in the home (as opposed to an institutionalized setting), and include bathing, dressing, grooming, toileting, transferring, feeding, and continence^30^. Assessing ADLs and IADLs captures additional information not obtained by accessing performance status alone. In one study, 23% of patients with adequate performance status were shown to have one or more deficits in IADLs^72^.

### Nutritional status

The incidence of malnutrition in the elderly population is very significant. Nutritional status should be assessed as part of GA, as malnutrition and weight are significant adverse factors in older patients and in patients with cancer. Although there is not one clear screening tool that is preferred, screening tools that have been used include body mass index (BMI), unintentional weight loss, or longer validated tools such as the Mini Nutrition Assessment (MNA)^38^. The MNA is well validated and correlates highly with clinical assessment and objective indicators of nutritional status and because of its validity in screening and assessing the risk of malnutrition, the MNA should be integrated in the GA^73^. Malnutrition is associated with treatment complications in patients receiving chemotherapy, radiation therapy, or surgery, and is associated with increased mortality^74^^-^^79^.

### Psychological status

Depression and psychological distress are common problems that impact patients with cancer and lead to poor quality of life, high caregiver burden, and functional decline. While studies have suggested that anxiety may decrease with aging, there is a consistent relationship between depression and increased age^80^. Depression is highly prevalent in older persons with cancer, with a range of 10%-65% across different GA studies^81^. Patients with cancer and depression are less likely to receive definitive treatment, and hence, experience worse survival compared to those without depression^82^. It may be necessary to also evaluate a patient with depression with fatigue, using a depression scale validated in elderly patients that relies less on somatic symptoms^83^. Brief screening tools may help clinicians in busy settings detect patients who are experiencing severe psychological distress. The distress thermometer (DT) is a single item that asks patients to rate their distress in the past week on a 0 (“no distress”) to 10 (“extreme distress”) scale^84^. It offers an efficient means of identifying advanced cancer patients with severe distress. It has been used in psycho-oncology and validated for patients and cancer patients’ families^85^.

### Association of geriatric assessment with outcomes

Over the last decade, GA has been integrated into oncology care and has contributed to uncover a substantial proportion of deficits in older cancer patients that would otherwise go unrecognized^81^. While results are difficult to compare, as different studies have used different components of GA, the most frequently assessed domains were functional status, comorbidity, depression, and cognition^86^. GA has been found to influence treatment decisions, which included reducing the intensity of chemotherapy, lower the amount of prescribed medications, or providing additional supportive care^26^. Because GA helps not only to better inform treatment decision-making but also helps to better tailor individualized treatment to an older patient who might otherwise be at greater toxicity risk, the recommendation of SIOG is that the findings from GA should be incorporated into oncology treatment decisions^26^. A Delphi technique was used to obtained consensus from an expert panel on the use of GA in clinical practice. The panel concluded that all patients aged 73 years or older should undergo GA and that all domains should be included in order to guide care processes^87^. However, no randomized controlled trial examining the effectiveness of GA in altering the treatment plan or improving outcomes for older adults with cancer has yet being published. In addition, few studies described the interventions that were carried out based on the results of the GA, nor how they impacted outcomes^86^^,^^88^. A prospective multicentric study on the large-scale feasibility and usefulness of GA in clinical oncology showed that GA detected unknown geriatric problems in 51% of patients ≥70 years old and when physicians became aware of the results, geriatric interventions and adapted treatment occurred in 25.7% and 25.3% of the patients, respectively^89^.

The choice of how comprehensive and detailed a GA should be may depend on the intended use. For risk stratification prior to chemotherapy, briefer tools based on GA may be more efficient in determining a patient’s predicted chemotoxicity risk. However, if the purpose of a GA is to identify conditions that put an older person at risk of toxicity and to intervene to decrease that risk prior to therapy, a more comprehensive evaluation may be warranted and helpful. One tool, the Cancer and Aging Research Group (CARG) score, was developed in a prospective multicenter cohort study of 500 patients ≥65 with cancer receiving chemotherapy. All patients underwent a GA that included measures of functional status, comorbidity, psychological state, social activity, social support, and nutrition. A predictive model was developed including GA variables along with patient demographic and clinical variables to predict grade 3 to 5 toxicity with chemotherapy administration (**Table 5**). Higher risk scores were associated with increased chemotoxicity (**Table 6**)^49^.

**Table 5:** CARG score to predict chemotherapy toxicity risk^49^

Risk factor	Score
Age ≥72 years	2
Cancer type (gastrointestinal or genitourinary)	2
Chemotherapy dosing, standard dose	2
Number of chemotherapy drugs, polychemotherapy	2
Hemoglobin (<11 g/dL in males) (<10 g/dL in females)	3
Creatinine clearance <34 mL/min (Jelliffe, ideal weight)	3
Hearing, fair or worse	2
Number of falls in the last 6 months, 1 or more	3
Taking medications with some help/unable	1
Walking 1 block, somewhat limited/limited a lot	2
Decreased social activity because of physical/emotional health problem, limited at least sometimes	1

**Table 6:** Chemotoxicity associated with CARG score^49^

Total risk score	Percentage of patients with grade 3-5 toxicity (%)
0-3	25
4-5	32
6-7	50
8-9	54
10-11	77
12-19	89

The Chemotherapy Risk Assessment Scale for High-Age Patients (CRASH) score was developed in a prospective, multicenter study among patients aged 70 and older receiving chemotherapy. GA variables were included along with patient clinical variables and chemotoxicity risk and predictive models were developed for grade 4 hematologic and for grade 3-4 non-hematologic toxicity (**Table 7**)^90^.

**Table 7:** The chemotherapy risk assessment scale for high-age patients (CRASH) score^90^

Predictors	Points		
	0	1	2
Hematologic score			
Diastolic blood pressure	≤72	>72	
IADLs	26-29	10-25	
LDH (if upper limit of normal 618 U/L, otherwise 0.74/L*ULN)	0-459		>459
Chemotoxicity	0-0.44	0.45-0.57	>0.57
Non-hematologic score			
ECOG performance status	0	1-2	3-4
Mini mental health status	30		<30
Mini Nutritional Assessment	28-30		<28
Chemotoxicity	0-0.44	0.45-0.57	>0.57

## Comprehensive geriatric assessment in the oncology clinic

The US National Comprehensive Cancer Network (NCCN) and SIOG have recommended that some form of geriatric assessment be conducted to help cancer specialists determine the best treatment for their older patients^26^. A panel with expertise in geriatric oncology performed a review of the literature and determined that GA can be valuable in oncology practice for following reasons: detection of impairment not identified in routine history or physical examination, ability to predict severe treatment-related toxicity, and ability to influence treatment choice and intensity. The panel recommended that the following domains be evaluated in a GA: functional status, comorbidity, cognition, mental health status, fatigue, social status and support, nutrition, and presence of geriatric syndromes. Although several combinations of tools and various models are available for implementation of GA in oncology practice, the expert panel could not endorse one over another^26^.

GA is time consuming and requires close cooperation between oncologists and geriatricians. An important practical aspect of GA is the feasibility of incorporating it into an already busy clinical oncology practice. Key considerations in performing the GA include the resources available (staff, space, and time), patient population (who will be assessed), what GA tools to use, and clinical follow-up (who will be responsible for using the GA results for the development of care plans and who will provide follow-up care). Important challenges in implementing GA in clinical practice include not having easy and timely access to geriatric expertise, patient burden of the additional hospital visits, and establishing collaboration between the GA team and oncologists regarding expectations of the population referred for GA and expected outcomes of the GA^91^.

A two-step approach has been suggested: the development of screening tools that would sort out who is an “older adult” with intact physiology and psychosocial conditions, and who is a vulnerable elder cancer patient in need of further multidisciplinary evaluation. Numerous geriatric screening tools have been developed and are increasingly implemented in daily practice. The most widely used screening instruments are the G8^92^, the abbreviated comprehensive geriatric assessment (aCGA)^93^, the Groningen frailty indicator (GFI)^94^ and the vulnerable elders survey-13 (VES-13)^95^. All of the frailty screening methods assess functional status and most also assess psychosocial functioning. The aCGA and G8 are the only methods designed specifically for assessment of frailty in elderly patients with cancer. VES-13 and G8 were evaluated for their utility to identify older allogenic hematopoietic cell transplant patients who are likely to have an abnormal GA or the presence of the frailty syndrome. Their findings suggested that G8 had a higher sensitivity and the VES-13 had a higher specificity. However, both screening tools had a modest negative predictive value to determine which patients were fit enough to bypass a full GA^96^. A recent review of frailty screening methods in older cancer patients showed that even in case of the highest sensitivity, the negative predictive value was only roughly 60% and this review suggested that, for now, it might be beneficial for all elderly patients with cancer to receive a complete geriatric assessment since available methods have insufficient discriminative power to select patients for further assessment^97^. A task force convened by SIOG conducted a systematic review of 17 different screening tests to determine which was more prognostic of an impaired CGA in older cancer patients. Across all studies, G8 was found to be more or equally sensitive than other instruments. They conclude that screening tools in older cancer patients should not replace GA. However in a busy clinical practice, the use of a screening tool is recommended to identify patients in need of further evaluation by GA. No specific tool was recommended or discouraged^98^.

## Information technology and GA in older cancer patients

Over the past 10 years, information and communication technology (ICT) within the healthcare system has evolved. Different domains of healthcare ICT include: mobile health (m-Health), telemedicine and telehealth, electronic medicine and electronic health (e-Medicine and e-Health)^99^. M-Health is defined as “medical and public health practice supported by mobile devices such as mobile phones, patient monitoring devices, and personal digital assistants (PDAs), and other wireless devices”^100^. Telemedicine is “the delivery of health services when there is a geographical separation between healthcare provider and patient or between healthcare providers”^99^. E-Medicine and e-Health provide solutions such as electronic medical record, and medical order entry system, as well as online platforms for patient and clinician training.

One of the most important components of GA and geriatric care is assessment of physical function. In addition to the electronic symptom gathering systems mentioned above, wearable devices and sensors can play a role in this field. For example, commercially available activity trackers can be used to assess older patients’ activity level. These trackers, mostly worn on the wrist, can track daily activities by measuring number of steps patients take on a daily basis. Although they differ in their accuracy in measuring activity, overall, most of them have high enough sensitivity and acceptable accuracy to be used in caring for older cancer patients^101^. Many older patients are at risk for falls. This risk may increase in cancer setting due to neurotoxic chemotherapy agents. Progress has been made to develop smartphone applications that automatically detect falls and is able to send request for help without user interaction^102^^-^^104^. Sleeping difficulty either due to circadian disruption or presence of symptoms such as pain, urinary frequency, or diarrhea is another important issue in cancer patients, leading to more fatigue. Efforts are being taken to allow for measuring sleep quality using smartphone applications, or wearable devices^105^.

While these innovations could change the field of geriatric oncology and the ways we assess and care for older cancer patients, we are still in the beginning of exploring these opportunities. In order to use these solutions effectively in geriatric assessment and cancer care, they need to be tested vigorously for their reliability and validity in the population of older patients with cancer. Moreover, the patients’ attitude toward technology and use of these devices should be taken into consideration. As these devices are being introduced into the healthcare system and research field, the privacy concerns should be addressed. It is essential to take into account the healthcare providers’ acceptability of using these devices in their practice. And finally, despite rapid pace of technology, a sound research methodology and design, which may limit the pace of the experiment, is needed to assess the impact of using these devices on outcome measures.

## Conclusion

GA is a critical process that can help to determine whether an older cancer patient is fit, vulnerable, or frail. It uncovers age-related conditions that should be addressed prior to or during cancer treatment with the goal of guiding care and reducing risks. The results of GA can be used to risk stratify patients, treat reversible conditions before cancer therapy, and guide cancer treatment decision-making. Oncologists should become familiar with the potential influence of patients’ difficulties uncovered by GA such as functional dependency, cognitive impairment or lack of social support on cancer treatment planning.

GA is time consuming and the number of geriatricians is scarce. Consequently, there is a need for screening procedures to determine which patients may benefit from GA. Yet, the effectiveness of such an approach—a screening tool for all older patients followed by an in-depth assessment of those deemed to be at risk—has not yet been established and validated by RCTs. Further studies are also needed in order to understand how to best manage elderly cancer patients with identified vulnerabilities.

Given the aging of the global population and the immense heterogeneity of physiologic age for patients of similar chronological age, the need for GA will likely increase in the coming years. Technology has great potential to make GA more feasible and efficient, as well as accessible for more oncologists in clinical settings.
